# Amplified fragment length polymorphism (AFLP) analysis of closely related wild and captive tsetse fly (*Glossina morsitans morsitans*) populations

**DOI:** 10.1186/1756-3305-3-47

**Published:** 2010-05-26

**Authors:** Gurdeep K Lall, Alistair C Darby, Bjorn Nystedt, Ewan T MacLeod, Richard P Bishop, Susan C Welburn

**Affiliations:** 1 Centre for Infectious Disease, School of Biomedical Sciences, The University of Edinburgh, Summerhall, Edinburgh, EH9 1QH, UK; 2 Centre for Genomic Research, University of Liverpool, Crown Street, Liverpool, L69 7ZB, UK; 3 Department of Molecular Evolution, Evolutionary Biology Center, Uppsala University, Norbyvägen 18 C, S-752 36 Uppsala, Sweden; 4 The Science for Life Laboratory, Karolinska Institutet Science Park, Tomtebodavägen 23 A, S-171 65 Solna, Sweden; 5 The International Livestock Research Institute (ILRI), PO Box 30709, Nairobi, Kenya

## Abstract

**Background:**

Tsetse flies (Diptera: Glossinidae) are vectors of trypanosomes that cause sleeping sickness in humans and nagana in livestock across sub-Saharan Africa. Tsetse control strategies rely on a detailed understanding of the epidemiology and ecology of tsetse together with genetic variation within and among populations. High-resolution nuclear genetic markers are useful tools for elucidation of the genetic basis of phenotypic traits. In this study amplified fragment length polymorphism (AFLP) markers were developed to analyze genetic variation in *Glossina morsitans morsitans *from laboratory and field-collected populations from Zimbabwe.

**Results:**

A total of seven hundred and fifty one loci from laboratory and field populations of *G. m. morsitans *from Zimbabwe were genotyped using AFLP with seven primer combinations. Analysis identified 335 polymorphic loci. The two populations could be distinguished by cluster and principal components analysis (PCA) analysis, indicating that AFLP markers can be used to separate genetically similar populations; at the same time differences observed between laboratory and field populations were not very great. Among the techniques investigated, the use of acetone was the most reliable method of preservation of tsetse for subsequent extraction of high molecular weight DNA. An interesting finding was that AFLP also enabled robust within-population discrimination of male and female tsetse flies due to their different X chromosome DNA complements.

**Conclusions:**

AFLP represents a useful additional tool to add to the suite of techniques currently available for the genetic analysis of tsetse populations and represents a useful resource for identification of the genetic basis of important phenotypic traits.

## Background

Over the past 30 years several techniques have been used to assess the genetic variation in populations of tsetse flies, these include protein polymorphisms in house-keeping genes or allozymes [1-4], microsatellites [5-7] and mitochondrial DNA techniques [4,6,8-10]. The markers used in these studies have provided useful information on phenotypic and genetic polymorphism in *Glossina *species. In particular they have indicated a degree of genetic differentiation between geographically separated tsetse populations, which was surprising, given the mobility of the flies combined with expectations derived from population genetics theory [11-13]. Mitochondrial variation is useful for discrimination of individual flies and reveals relatively high levels of haplotype divergence between randomly chosen individuals among four species analysed, including *G. morsitans *[11]. However, arthropod mitochondrial genomes are small in size (14-25 kilobases) and maternally inherited [14], hence data interpretation differs from that of variation in nuclear genes.

The number of tsetse loci in the nuclear genome using the techniques sampled for variation to date is relatively limited, for example in *Glossina morsitans morsitans *a total of 45 allozymes and five microsatellites were reported as being analysed [11]. To provide useful genetic markers for association with phenotpyes of interest, such as vector competence, higher resolution coverage is required for *Glossina *species, whose estimated genome sizes range from 600 Mbp for *G. morsitans *to 7,000 Mbp for *G. palpalis*, the latter being considerably larger than the human genome [15]. Amplified Fragment Length polymorphism (AFLP) is a fingerprinting technique that is based on selective PCR amplification of restriction fragments from a total digest of genomic DNA [16]. It represents a powerful genotyping method that increases the utility of simple restriction enzyme digestion analysis by combining this with PCR and can typically sample 50-100 restriction enzyme fragments on a single polyacrylamide gel. Since AFLP samples polymorphism at hundreds of independent nuclear loci without any requirement for prior sequence information [16] the technique typically provides an in depth assessment of genome-wide variation [17]. By contrast restriction fragment length polymorphisms (RFLPs) are restricted to sampling one locus at a time and therefore have much lower throughput than AFLPs.

The AFLP technique is a convenient and reliable tool with the capacity to discriminate closely related populations, and has been successfully applied to mosquito species and populations for genetic mapping, genotype identification, taxonomic and population genetic studies [18].

Population genetic studies of insect vectors such as tsetse flies requires the collection of a large number of field caught insects from remote rural locations, followed by DNA extraction and analysis. For accurate molecular analysis to be performed, it is crucial to generate DNA of good quality. It is therefore essential to identify suitable methods for maintaining the integrity of the DNA samples after collection, until extraction and analysis are performed. Tsetse flies collected for genetic analyses have in previous studies been stored in liquid nitrogen or under conditions of ultra-low temperature refrigeration [9,19-21]. However, liquid nitrogen facilities and reliable power sources for freezer storage of materials are rarely available in areas where tsetse flies are collected and more convenient and cheaper alternative methods to preserve samples for DNA analysis are required.

The primary objective of this work was to investigate the use of AFLP as markers of genetic variation in tsetse populations, by comparing the genetic profiles of a laboratory colony, to that of a field-collected *Glossina morsitans morsitans *population. Multiple primer pairs were used to maximize the number of loci allowing the selection of optimal primer sets; in addition we aimed to identify a suitably inexpensive medium for storage of field-collected tsetse flies that would preserve the molecular integrity of flies so that high quality DNA could be retrieved for subsequent molecular analysis.

## Results

### DNA Quality

Electrophoresis of DNA from freshly killed flies exhibited a distinct band of high molecular weight (HMW) DNA with no low molecular weight (LMW) DNA visible. Acetone preserved flies showed a strong HMW DNA band with a limited amount of LMW DNA smearing. Flies stored in lysis buffer or ethanol-preserved flies showed a weak HMW DNA band with extensive LMW DNA smearing. Flies stored in SDS buffer or stored on FTA card matrix showed DNA profiles with LMW DNA appearing as a smear, HMW DNA band was rarely observed.

In general, electrophoresis of DNA from flies dried prior to preservation demonstrated inferior HMW DNA bands compared to freshly preserved flies. Although HMW DNA bands were detected, there was also extensive smearing in the LMW region of the gel.

### DNA Yield and Purity

**Figure 1:**
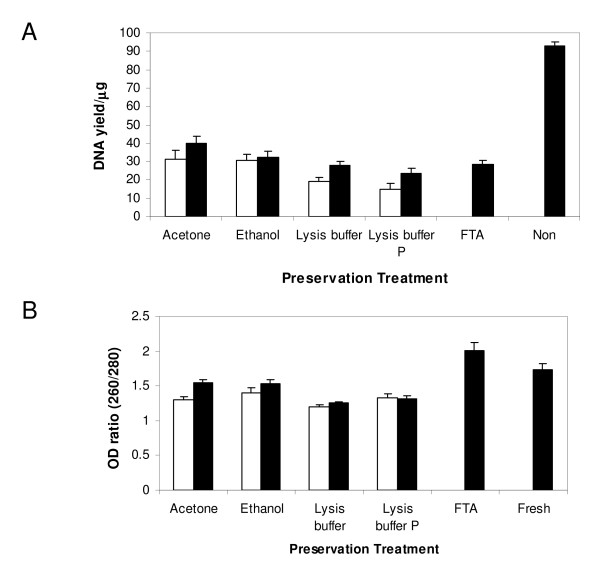
**Mean yield (a) and mean purity (b) of DNA extracted from tsetse stored using different preservation techniques**. White columns show samples dried before preservation, black columns show samples preserved fresh (FTA and fresh samples were not dried before preservation). Error bars show the standard error of the mean.

The overall mean yield of DNA from preserved material (shown in Figure 1a) was significantly lower (mean 28.4 μg, standard error of mean (SE) ± 1.25) and around three times less than of DNA from fresh material (mean 93.2 μg SE ± 1.96; T = -18.55 d.f.= 82 p > 0.001). Similarly, the 260/280 nm optical density (OD) purity ratio of preserved samples (Figure 1b) was significantly lower than that isolated from fresh material (mean OD ratio: preserved 1.46 SE ± 0.03; fresh 1.74 SE ± 0.08; T = -2.82073 d.f.= 82 p = 0.003). The fresh samples were not included in subsequent analyses.

The preservation technique used had a significant effect on the DNA yield obtained and (GLM yield versus treatment, dried/undried: F_3,63 _= 9.88 p < 0.001) 260/280 nm OD ratios (GLM OD ratio versus treatment, dried/undried: F_3,63 _= 10.41 p < 0.001). Samples that were dried prior to preservation showed significantly reduced yields (GLM yield versus treatment, dried/undried: F_1,63 _= 8.68 p = 0.005) and 260/280 nm OD ratios (GLM OD ratio versus treatment, dried/undried: F_1,63 _= 9.8 p = 0.003) compared to freshly preserved flies. Tukey-Kramer's analysis did not demonstrate any significant difference in DNA yields or purity between acetone and ethanol treatments.

### AFLP genotype analysis

A total of 129 *G. m. morsitans *flies were analyzed comprising 59 laboratory and 70 from a wild Zimbabwean tsetse population. A total of 751 loci over both populations were scored using seven primer combinations and 45% (335/751) were polymorphic. Popgene analysis of polymorphic alleles is summarized in Table 1. The partitioning of molecular variance within and between the two populations calculated by AMOVA showed that 9.41% of the molecular variance was between the two populations and 90.59% was within the populations. The total genetic diversity (Ht) across the two populations was 0.21 +/- 0.03 and within-population gene diversity (Hs) was 0.20 +/- 0.03.

**Table 1:** Genetic variability of the tsetse populations measured by the observed number of polymorphic loci, gene diversity (heterozygosity, H) and the degree of genetic differentiation among populations (Gst).

Population	Number of individuals	Percentage of polymorphic loci	**Nei's **[24]** gene diversity h**	G_ST_
Both	129	44.61 (335/751)	0.21 ± 0.17	0.0386
Lab	59	84.18 (282/398)	0.21 ± 0.18	
Field	70	92.54 (310/362)	0.19 ± 0.17	

597 AFLP bands from 129 individuals using 7 primer combinations.

### Cluster and PCA Analysis

**Figure 2:**
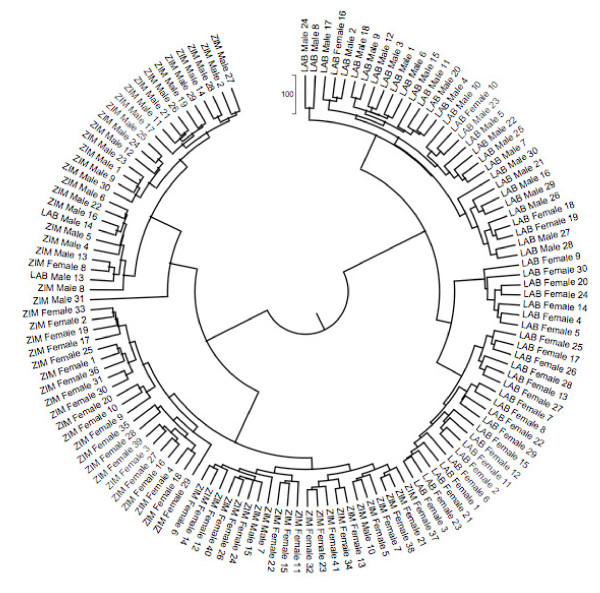
**AFLP genotype hierarchical cluster analysis of male and female *G. morsitans *from laboratory and field populations**. Using Ward's minimum variance method with pairwise Manhattan distances between individuals. AFLP bands from seven primer primers were used (Table 2), 597 AFLP bands from 129 individuals. Label prefixes LAB and ZIM denote laboratory and Zimbabwe samples respectively, followed by fly sex and sample number.

**Figure 3:**
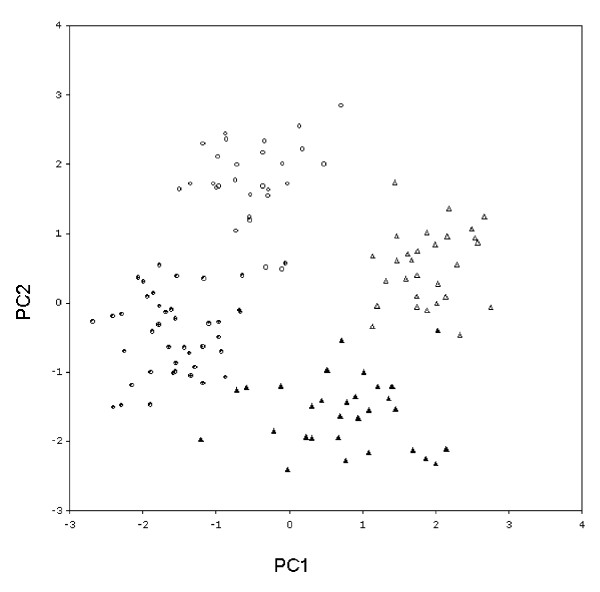
**Principal component analysis of the first (PC1) and second (PC2) components showing discrete clustering of Zimbabwe field collected *G. m. morsitans *(circle) and laboratory reared flies (triangle) demonstrating distinct population genotypes and robust within population separation of male (hollow shape) and female (filled shape) tsetse**. AFLP bands from 129 individual insects using seven primer pair combinations (see Table 2) comprising 597 AFLP bands were analyzed.

Both cluster and PCA methods showed that individuals from the same population group together and were distinct from individuals from the other population (Figures 2 and 3). In each population there was also a grouping of male and female samples. There are few exceptions to the groups described.

## Discussion

Estimates of gene flow between tsetse populations based on population genetic analyses are important in the context of predicting the long-term effectiveness of tsetse control measures [12]. However, genetic markers also have other uses, in particular as tools that can assist in determining the genetic basis underpinning important phenotypes. When captive colonies are used to analyse phenotpyes such as factors influencing vector competence [22,23] it is important that this can be related back to the field populations.

This study demonstrates that AFLPs can be used to generate high-resolution genetic markers in natural populations of *G. m. morsitans*. Two distinct population groups were apparent from the cluster and PCA analysis (Figures 2 and 3). Although the separation of the two populations is not perfect, the results demonstrate that the AFLP markers can be used to separate two genetically close populations. The level of Nei's genetic diversity [24], h was small in both populations and the amount of genetic variation detected in the laboratory and a Zimbabwean population was similar. In addition, the GST value of 0.0386 indicates that there is little genetic differentiation between the natural Zimbabwean and the laboratory population. The results in the current study suggest that the establishment and long term maintenance of the insect colony process (> 30 years) has resulted in little effect on genetic variation as estimated by AFLPs. The fact that the flies from the laboratory colony exhibit only limited divergence from the field populations is encouraging for extrapolation of experimental results to the 'real world'. The field population did, however, show a higher number of loci and a higher percentage of polymorphic loci than the laboratory population (Table 1). AMOVA results showed that the populations are closely related as there was more variation within (91%) the populations than between (9%) them. This result, derived from variation in nuclear genes, is in agreement with that obtained for *G. morsitan morsitans *using mitochondrial markers [11]. It was interesting that males and females were clearly separated using the AFLP analysis, however this can be explained by the greater DNA content of female flies where the dosage of X determines sex. Hence males are XY or XYY whereas females can be XX, XXY or XXXY [25]. The identification of markers that can discriminate males and female flies could be valuable in the future in the identification of sex ratio distortion genes [25] in the tsetse genome.

Whenever possible it is preferable for DNA to be extracted from fresh tsetse material. However, when this is impractical due to location, cost or a combination of the two, the present results suggest that acetone should be used in preference to the other preservatives tested. Flies preserved in acetone gave the highest quality HMW DNA and resulted in DNA of good quality on both fresh and pre-dried material. Ethanol has been widely used for the preservation of various biological samples including insects [26-29]. In the current work both tsetse DNA yield and purity from ethanol samples were similar to acetone preserved material (Figure 1). However, ethanol preserved samples contained a low amount of HMW DNA and ethanol was inferior in the preservation of fresh flies (Figure 1). This observation is consistent with previous studies that demonstrated poor ethanol preservation in the presence of water [26,28]. This problem was not overcome by using 100% proof ethanol and changing the ethanol after 24 h (this study).

Although the material preserved in SDS lysis buffers (either with or without proteinase K) or on a FTA card showed reasonable DNA yield and purity in all cases it was degraded LMW DNA with pronounced smearing that predominated. The LMW smearing was most extensive in the FTA samples suggesting that FTA cards are not appropriate media for storing large insects. This may be because not all parts of the fly were in contact with the matrix, allowing endonucleases activity resulting in DNA degradation.

The data presented here shows that acetone was a superior medium for preserving tsetse DNA. An additional advantage is that acetone is used as a bait component in tsetse traps and is therefore readily available to field workers collecting tsetse flies. The DNA obtained using this method could be used for PCR of specific genes, microsatellite analysis, AFLP and other molecular techniques that require high quality DNA. In addition, acetone may be an appropriate storage media for other important dipteran vectors, such as mosquitoes due to its high penetrability and dehydrating abilities. However, one of the disadvantages of using acetone is that many aircraft companies will not allow acetone to be carried in the hold.

The relative value of a range of genetic markers, specifically Random Amplification of Polymorphic DNA (RAPDs), Inter-simple-sequence repeat PCR and microsatellites as applied to the analysis of variation between strains of the silkworm (*Bombyx mori*) has been reviewed [30], although this study did not include AFLPs. The basic conclusion was that all techniques are useful and have different strengths and weaknesses in terms of factors such as resolution, ease of use and cost. As regards nuclear genetic markers in tsetse flies, allozymes sample approximately 50 loci and do reveal a degree of inter-population diversity, however they are not selectively neutral and hence may not always accurately reflect population history. Microsatellites reveal significant allelic polymorphism but are relatively few in number and are limited by the requirement for sequence data for primer design. Both have proved useful for initial analysis of inter-population differentiation in the field [11,12]. The initial AFLP study described here has identified numerous previously unknown polymorphic loci in *G. m. morsitans *which as indicated above will have value in areas of tsetse research, additional to differentiation of field populations. Because these loci are essentially randomly distributed [16], greater in number and not necessarily selected for rapid evolution, AFLP analysis may provide a more accurate assessment of genome-wide differentiation in *Glossina*. In future it should prove possible to generate microsatellite markers more rapidly as expressed sequence tag and genome sequence databases [31] are systematically searched for variable number tandem repeat (VNTR) and single nucleotide polymorphisms (SNP). However, due to the large size of *Glossina *genomes [15] it seems likely that not all of these will have genome sequences available in the immediate future. AFLP markers which can generated over a wide dynamic range of genome sizes [16] should continue to be of value.

## Conclusions

This study demonstrates the usefulness of AFLP markers in tsetse flies to assess relationships between and within tsetse populations and their potential as genetic markers for identification of the genetic basis of phenotypes. AFLP markers based on sampling hundreds of polymorphic loci are able to define differences even between closely related populations. As such, they may provide a particularly accurate technique for analysis of genetic variation and represent an additional tool for exploring the genetic diversity of tsetse populations. In the medium term application of such data this will be helpful in designing, evaluating and implementing effective tsetse control strategies to prevent trypanosomiasis.

## Methods

### Insect material

*G. m. morsitans *(Westwood) originally isolated from Kariba, Zimbabwe [32], were obtained from long-term colonies maintained at the University of Edinburgh. This colony was set up from individuals obtained from the University of Bristol in 1996 at the University of Glasgow. In 1999 the colony from the University of Glasgow was moved to the University of Edinburgh. Since 1996 the population has fluctuated in size but the numbers have maintained between 1000 to 2500 breeding females since 2000. *Glossina morsitans centralis*, which was used in initial investigations to examine primer pair combinations, were obtained from the long-term colonies maintained at the International Livestock Research Institute, Nairobi, Kenya. Field collected Zimbabwean *G. m. morsitans *were obtained from the Rekomitjie Research Station in the Zambezi valley, near Chiuyi River, Zimbabwe (16 deg 23 min 55 s S, 29 deg 23 min 11 s E) and caught using Epsilon traps baited with acetone (500 mg/h), octenol (0.5 mg/h), 4-methylphenol (1 mg/h) and 3-n-proylphenol (0.1 mg/h). Traps were set at intervals of about 300 m and were emptied daily.

#### Insect material for DNA preservation

Eighty-four, colony reared *G. m. morsitans *were collected 20 days post-eclosion. Ten insects were used as fresh, non-preserved controls. Of the remaining 74 flies; 10 were crushed on Whatman FTA preservation matrix (Bioline Ltd., London, UK); 10 were preserved in each of the four preservation media. The preservation media used were (i) pure grade absolute acetone (99.5%); (ii) pure grade absolute ethanol (Fisher Scientific Ltd., Loughborough, UK), (iii) lysis buffer (500 mM Tris-HCl at pH 9.2, 250 mM ethylenediaminetetra-acetic acid (EDTA) and (iv) 2.5% w/v sodium dodecyl sulfate (SDS) and lysis buffer with proteinase K (50 μg/ml). Lysis buffer with proteinase K is hereafter referred to as lysis buffer P. Unless otherwise stated all chemicals and enzymes were purchased from Sigma-Aldrich, Gillingham, UK.

Field caught materials are regularly exposed to desiccation and ultraviolet radiation before collection and to simulate field conditions, 24 flies were air dried in the sun for 12 h before six were preserved in each of the four preservation media. All flies were stored individually in two ml of preservative for two months at room temperature. In the case of ethanol- and acetone-preserved samples, preservatives were replaced after the initial 24 h to ensure complete dehydration of samples.

#### Insect material for AFLP analysis

Fifty-nine laboratory reared *G. m. morsitans *and 70 field collected Zimbabwean *G. m. morsitans *flies were used in this study. All field-collected samples were preserved in acetone.

### DNA Extraction

Flies were removed from the storage media and excess preservative removed by blotting on to absorbent paper. Whole insects were used for extraction, using a cetyltrimethylammonium bromide (CTAB) extraction method [33]. DNA from freshly killed controls and FTA-preserved flies was extracted in the same way. Following extraction, the genomic DNA was dissolved in 50 μl of distilled water [33]. DNA for AFLP analysis was quantified by spectrophotometry (Beckman Coulter DU 530) and diluted to a working concentration of 500 ng/μl.

### DNA Quantity and Quality

Genomic DNA was subjected to spectrophotometry (Beckman Coulter DU 530) to determine yield and purity. DNA yields were calculated from concentration values while purity was determined by the 260 nm/280 nm absorbance ratio readings [34]. Quality was accessed by running 500 ng of each sample on a 1.5% agarose gel in 1 X TBE buffer at 150 v for 30 min. The gel was stained with ethidium bromide (1 μg/ml) and bands were observed and photographed using an ultraviolet transilluminator linked to a Gel Doc 2000 BioRad system (BioRad, Hemel Hempstead, UK).

### AFLP genotyping

AFLP reactions were performed using the AFLP genome Plant Mapping Kit (Perkin Elmer Applied Biosystems, Warrington, UK) following the method of De Vos *et al*. [16]. All adaptors and PCR primers were provided as kit components. In brief, genomic DNA was digested with EcoRI and MseI restriction enzymes. The DNA fragments were then ligated with EcoRI and MseI adaptors, generating template DNA for pre-selective PCR amplification.

The pre-selective amplification thermal cycling conditions were: an initial hold at 72°C for 2 min, followed by 20 cycles of 20 s at 94°C, 30 s at 56°C, and 2 min at 72°C with a final 30 min hold at 60°C. Following pre-selective amplification using the primer sets supplied with the kit, template for selective amplification was prepared by diluting 10 μl of each of the pre-selective amplification products with 190 μl TE buffer. Selective amplification was performed using combinations of EcoR1 and Mse1 primer pairs. Selective amplification thermal cycling conditions were: an initial denaturation at 94°C for 2 min, followed by 30 PCR cycles of 20 s at 94°C, 30 s at 66°C (10 cycles with a 1°C reduction at every cycle), 56°C (subsequent 20 cycles), and 2 min at 72°C and a final 30 min hold at 60°C.

The primer combinations were selected by testing all 64 possible primer combinations (8 Mse1 and 8 EcoR1) on males and females from *G. m. morsitans *and *G. m. centralis*. Ten primer combinations gave banding patterns (Table 2) and seven primer combinations were selected that produced long profiles (alleles of 50-500 bp) with evidence of polymorphisms (see Table 1).

**Table 2:** Suitable AFLP primer combinations for *G. morsitans *DNA.

	Mse1 primer tail							
	
EcoR1 primer tail	-CAA	-CAC	-CAG	-CAT	-CTA	-CTC	-CTG	-CTT
-AAC								

-AAG	+	**++**						

-ACA	**++**					**++**		**++**

-ACC								

-ACG								

-ACT	**++**						**++**	

-AGC	+			**++**				

-AGG			+					

**+ **indicates compatible primer pair; ++ suitable primer used for analysis in this study.

AFLP alleles were detected on an ABI 3700 automatic DNA sequencer (Perkin Elmer Applied Biosystems, Beaconsfield, UK), and the AFLP allele sizes were measured using GeneMapper 3.7 software (Applied Biosystems). Binary data tables showing alleles were generated and imported into tab delimited text files. Analysis of the number, percentages of polymorphic loci and gene diversities within and across populations were calculated in Popgene [35]. Popgene generates two estimates of heterozygosity, firstly Nei's [24] heterozygosity and secondly the expected heterozygosity using the algorithm of Leven [36] which gives the same result as Nei's [37] unbiased heterozygosity. Partitioning of molecular variance within and between populations was calculated by analysis of molecular variance (AMOVA) using Arlequin software [38].

### Statistical Analysis

Differences in DNA yield and purity based on the OD 260/280 nm ratio between fresh and preserved flies were analyzed using t-tests. General linear model (GLM) analysis was performed to investigate the effect of preservative and drying on DNA yield and 260/280 nm ratio. All analyses were performed with MINITAB version 14 (Minitab Ltd., Coventry UK).

Principal components analysis (PCA) and hierarchical cluster analysis were performed in R (Version 2.2.1) [39]. Prior to the analysis, one outlier (ZIM Male 18) was excluded based on visual inspection, and the dataset was pruned to remove all non-informative alleles. Non-informative sites were defined as alleles present in all or none of the included individuals. PCA was performed on centered, non-scaled data and the hierarchical cluster analysis used Ward's minimum variance method with pairwise Manhattan distances between individuals.

## Competing interests

The authors declare that they have no competing interests.

## Authors' contributions

GKL was involved in the design of the study, carried out the molecular experiments, analysed the data and drafted the paper. ACD was involved in the design of the study and drafted the paper. BN analysed the data and drafted the paper. ETM drafted the paper. RB was involved in the design of the study and drafted the paper. SCW was involved in the design of the study and drafted the paper. All authors read and approved the final manuscript.
